# Contrast Extravasation Post Thrombectomy in Patients With Acute Cerebral Stroke: A Review and Recommendations for Future Studies

**DOI:** 10.7759/cureus.10616

**Published:** 2020-09-23

**Authors:** Eric Whitney, Yasir R Khan, Anthony Alastra, Michael Schiraldi, Javed Siddiqi

**Affiliations:** 1 Neurosurgery, Desert Regional Medical Center, Palm Springs, USA; 2 Neurosurgery, Redlands Community Hospital, Redlands, USA; 3 Neurosurgery, Riverside University Health System Medical Center, Moreno Valley, USA; 4 Neurosurgery, Arrowhead Regional Medical Center, Colton, USA; 5 Neurosurgery, California University of Science and Medicine, Colton, USA

**Keywords:** angiographic blush, contrast extravasation, mechanical thrombectomy, stroke, large vessel occlusion

## Abstract

Mechanical thrombectomy (MT) for cerebral revascularization in acute stroke is now considered standard of care in select patients. Patients are assessed routinely after MT with CT scanning. The phenomenon of contrast staining is well documented in the literature and is posited to be related to increased blood-brain barrier (BBB) permeability of susceptible and/or infarcting brain tissue allowing angiographic contrast to be visualized outside the normal cerebral vasculature. In some cases, this can progress to include frank blood/contrast extravasation or even more seriously lead to intraparenchymal hemorrhage (IPH) with less favorable clinical outcomes. The relationship of this staining phenomenon and how it may have a cause or effect relationship with progression to hemorrhage is unclear. Many studies have been performed trying to better characterize this radiographic finding in terms of accurate diagnosis and potential for influencing prognosis. A literature review included a glaring lack of standardization in the application of terminology and quantitative/qualitative analysis. Dual energy CT (DECT) appears to be the best imaging modality to differentiate blood from contrast, but its application is limited since it is not as available as conventional CT. The possibility that risk factors are associated with progression of mixed density (blood and contrast) extravasations to frank IPH with resultant poorer outcomes is suggested in some studies. Overall, there remains a lack of consensus on how to best interpret this radiographic finding in altering any future stroke treatment(s). Recommendations of how to overcome this are postulated by the authors, which include standardization of terminology, progression toward more DECT use.

## Introduction and background

Mechanical thrombectomy (MT) is a procedure where catheters are guided through a patient's arteries to the site where the blood clot is in the brain. Once positioned, a combination of stent retrievers and catheter aspiration is utilized to remove or disrupt the thrombus. Various contrast agents are used to enhance the visibility of vessels during angiography. After an intervention, some patients undergo non-contrast head CT that reveal hyperdensities. These are either due to contrast extravasation or hemorrhage. These hyperdensities are reported in 31.2% to 87.5% of patients where 46% reported hemorrhage [1]. Accurate interpretation, understanding their clinical significance, screening those at risk for complications, and developing preventative measures have been the topic of many studies. This paper is a review of the existing literature to date on the topic of contrast extravasation after thrombectomy and a discussion regarding the current understanding of its significance, proposals for standardization of terminology, as well as implications for future investigations.

## Review

Current understanding

Terminology

**Figure 1:**
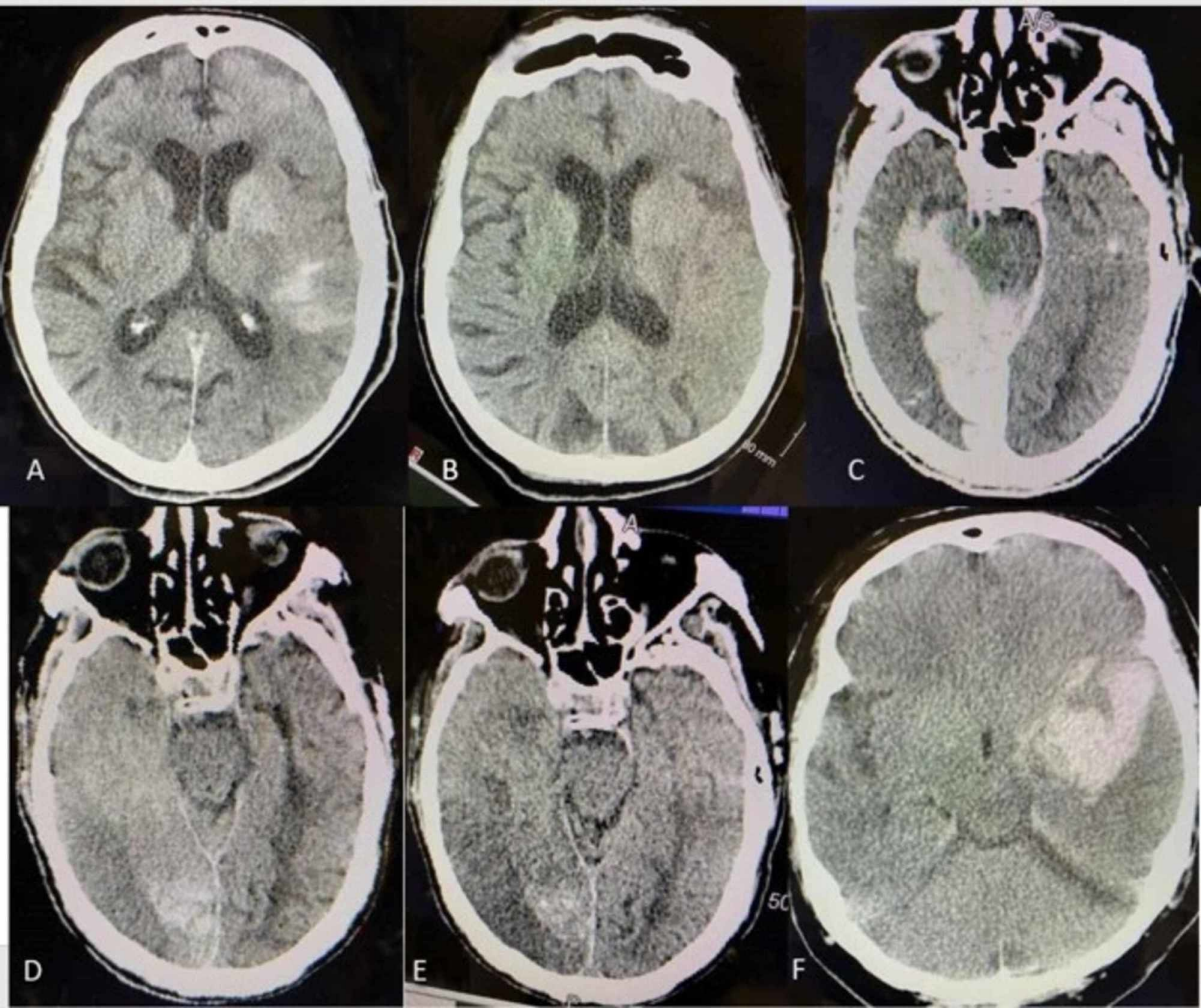
(A) and (B) show pure contrast staining, with (A) showing staining directly after thrombectomy and (B) 12 hrs later. (C), (D), and (E) are mixed contrast and blood, with (C) being after thrombectomy, (D) being 24 hrs after thrombectomy, and (E) being 3 days after thrombectomy. (F) is mostly blood (IPH), representing hematoma, that expanded and required decompressive craniectomy and hematoma evacuation 24 hrs later. IPH: intraparenchymal hemorrhage.

Intracranial hemorrhage after reperfusion therapy is a complication that can be difficult to interpret by additional hyperdensity on CT created by contrast and not just the presence of blood. This extravasation is the result of the disruption of the blood-brain barrier (BBB) due to ischemia with increased permeability [2]. Contrast is neurotoxic and weakens the BBB further when extravasation occurs [2]. If only the endothelial cell is damaged, the hyperdensity may represent contrast without blood. However, when the basal lamina is damaged the hyperdensity is likely hemorrhage or mixed density including contrast [3]. Contrast affects brain imaging for at least 24 hours (hrs) until cleared [2]. However, blood undergoes degradation and is seen on imaging. Studies listed in Table 1 have focused on differentiating the source of CT hyperdensity, exploring various imaging techniques, and understanding clinical outcomes. Accurate hyperdensity interpretation leads to improved interventions and prognosis. However, terms such as contrast extravasation, hyperdensity, contrast enhancement, metallic hyperdensity, and contrast staining are used interchangeably (Figure 1).

**Table 1:** Terms used in different studies and their conclusions AIS: androgen insensitivity syndrome; CEn: contrast ent; CEx: contrast extravasation; CSt: contrast staining; DECT: dual-energy CT; DSA: digital subtraction angiography; HA: hyperattenuation; HD: hyperdensity; hrs: hours; IAT: intra-arterial thrombectomy; IOM: iodine overlay map; IPH: intraparenchymal hemorrhage; MCI: microcatheter contrast injections; MHD: metallic hyperdensity sign; MT: mechanical thrombectomy; PCS: posterior circulation stroke; sIPH: symptomatic intraparenchymal hemorrhage; VNC: virtual noncontrast-enhanced.

Study	Term	First scan post MT	Repeat scan	Definition of term	Conclusion
Sun et al. 2019 [6] n = 108	CEx	DECT w/in 24 hrs	CT w/in 72 hrs	HA seen only on IOM	"CEx was an independent and strong predictor of poor outcome of unfavorable clinical outcomes."
An et al. 2019 [7] n = 180	CSt	DECT 12-24 hrs	CT w/in 72 hrs	HA seen only on IOM	CSt "seems to have no effect on functional outcome and sICH."
Cabral et al. 2017 [8] n = 71	HA lesion & CSt	Immediate CT	CT at 24 hrs	HD no longer visible after 24 hrs on CT	"HA lesions ... may predict final infarct."
Yedavalli et al. 2017 [2] n = 10	CEx	CT w/in 24 hrs	MRI w/in 48 hrs then CT at 72 hrs	Less than 50 or greater than 90 HU on CT	CT or MRI "should be done both after 72 hrs for confirmation."
Payabvash et al. 2015 [9] n = 80	CEx	CT w/in 12 hrs	CT w/in 12-36 hrs	HD no longer visible on 24 hrs CT. No rim of hypoattenuation.	"Higher residual contrast stagnation in affected MCA is associated w/ increased risk of IPH."
Song et al. 2015 [10] n = 39	CEn	Immediate CT	CT at 24 hrs	HD no longer visible on 24 hrs CT	CEn "on NECT scans obtained immediately after IAT could be predictive of malignant cerebral edema."
Lummel et al. 2014 [11] n = 101	CEn	Immediate post MT	CT at 24 hrs	HD no longer visible on 24 hrs CT	HD "seems not to be of any prognostic value regarding clinical outcome."
Amans et al. 2014 [5] n = 36	CSt	Within 72 hrs	Delayed	HU > 40. Respected anatomic boundaries. No mass effect and no peripheral edema.	CSt "on CT after DSA in AIS patients was likely to infarct and unlikely to hemorrhage."
Payabvash et al. 2014 [3] n = 135	CEx	Immediate post MT	CT at 24 hrs	HD no longer visible on 24 hrs CT. Without mass effect or hypoattenuating rim.	"HU < 50 of the most visibly hyperattenuating HD ... was specific differentiating CEx from IPH."
Phan et al. 2012 [12] n = 40	CEx & CSt	Immediate DECT	CT or MRI 24-48 hrs	No distinction in terms was made HA seen only on IOM. Follow-up image where HD no longer visible.	"DECT can accurately differentiate all types of ICH from iodinated contrast."
Antonucci et al. 2012 [13] n = 3	CEn	Immediate CT	CT at 24 hrs	HD no longer visible on 24 hrs CT	"CEn demarcated areas of complete cerebral infarction."
Khatri 2010 [14] n = 77	CEx CEn	Immediate CT	CT at 24 hrs	CEx: HU > 90 seen at and after 24 hrs CEn: HD no longer visible on 24 hrs CT	"MCI was assoc w/ ICH."
Nakano et al. 2006 [15] n = 61	CSt & CEn & CEx	Immediate CT	CT at 24 hrs and then again 3-7 days	No distinction in terms was made. HD no longer visible on 24 hrs CT.	"Cortical effacement may be an advanced CT sign with BBB disruption and potential risk for hemorrhagic transformations."
Jang et al. 2006 [16] n = 94	HD variety	Immediate CT	CT at 24 hrs MR 3-5 days	Contrast: If no longer visible on any image 24 hrs after initial scan	"Most of the soft HD lesions were benign, and although all of the metallic HD lesions were hemorrhagic."
Yoon et al. 2004 [4] n = 62	CEn CEx	Immediate CT	CT at 24 hrs	CEn: HD no longer visible on 24 hrs CT CEx: HU > 90 visible on 24 hrs CT	CEx "is highly associated with parenchymatous hematoma and should be considered a negative prognostic sign."
Nakano et al. 2001 [17] n = 77	CEx	Immediate CT	CT at 24 hrs CT 3-7 days	HD no longer visible on 24 hrs CT	"Hyperdense areas were a significant risk factor for severe hemorrhagic transformations."
Mericle et al. 2000 [18] n = 27	CEx	Immediate CT	CT at 24 hrs	HD no longer visible at 24 hrs and/or HU > 90	"This grading system should prove useful as a preliminary guide for predicting outcomes of patients with CEx."

In 2004, Yoon et al. sought to establish clear terminology distinguishing between contrast enhancement and contrast extravasation, noting that the later resulted in worse clinical outcomes. Contrast enhancement was defined as rapid clearance (within 24 hrs without residual mass effect). Contrast extravasation was defined as maximal Hounsfield units (HU) > 90 and included a mixture of contrast/blood with residual mass effect, persistence on follow-up CT, and was linked to higher rates of hemorrhagic transformation [4]. While the distinction between these findings may be important, most literature reviewed did not differentiate between the two. In fact, Payabvash et al. believed that distinguishing between the two on CT is not possible and felt to be clinically irrelevant [3].

Additional ambiguity occurred in 2014 when Amans et al. used the term ‘contrast staining’ for hyperdensities with HU greater than 40, stayed within normal anatomic boundaries, lacked edema, and were without mass effect. However, the exact timing of post-procedure CT was unclear because the inclusion criteria were imaging within 72 hrs [5]. Therefore, based on other studies, any image with hyperdensity persisting longer than 24 hrs would be classified as hemorrhage. Table 1 presents, in chronological order, the terms used in each study, how they were defined, and what conclusion was drawn.

Contrast vs Hemorrhage: HU, Timing, Anatomic Location

In 2014, Payabvash et al. reviewed imaging on 135 patients and defined hyperdensities persisting longer than 24 hrs and/or create mass effect with a hypoattenuation rim as a hemorrhage and lesions that disappeared within 24 hrs with no mass effect and/or edema as contrast extravasation. They concluded that a hyperattenuating hyperdensity with Hounsfield units (HU) less than 50 was contrast extravasation. For those patients with hyperdensities greater than 50 HU, contrast extravasation could not be ruled out. Hyperdensities, 39-57% of the time, represented contrast extravasation [3]. Yedavalli used 50 to 90 HU to classify hemorrhage and anything outside that range as contrast extravasation [2]. While terminology vacillated, the clearest parameter established for distinguishing between blood and contrast was time. If after 24 hrs there was visible hyperdensity on CT, it was considered hemorrhage [3].

Xu et al. conducted a retrospective study on 59 patients who had ‘metallic hyperdensity sign’ defined as a diameter greater than 1 cm in the basal ganglia and HU greater than 90 and concluded, with 90.5% specificity and a negative predictive value of 95.7%, conversion to parenchymal hemorrhage [19].

Imaging Technology

CT, flat-panel CT, dual-energy CT (DECT), MRI, and angiography were all evaluated in attempts to accurately determine the source of post-thrombectomy CT hyperdensities. Conventional CT is the mainstay imaging modality in the majority of the literature due to its widespread availability. MRI is costly and takes longer to acquire interpretable images. A flat-panel CT allows for larger volume per rotation and higher spatial resolution when compared to traditional CT. DECT uses two different x-ray energy levels to acquire images, as compared to standard CT that only uses one.

Yedavalli et al. investigated the prevalence of diagnostic complications of hyperdensities seen on CT post thrombectomy and the interpretation of the hyperdensity, which when associated with hemorrhage leads to an alteration in clinical management. They concluded that hyperintensities detected on MRI performed within the first 48 hrs can lead to false-positive results of hemorrhage and advocated for post-thrombectomy MRI at 72 hrs. However, their study only looked at T1 and T2 images and did not specify the type of MR scanner used [2]. You et al., utilizing a 3T MRI immediately after mechanical thrombectomy, concluded that diffusion-weighted imaging (DWI) and gradient-recalled echo (GRE) protocols could differentiate between contrast staining and hemorrhagic transformation [20].

Payabvash et al. concluded the absence of hyperdensity on flat-panel CT immediately after thrombectomy could exclude intraparenchymal hemorrhage as it is a higher quality image [21]. However, this same conclusion cannot be made with conventional CT as Payabvash et al. also described six out of 61 patients developed intraparenchymal hemorrhage one to five days after initial CT [3]. CT perfusion studies demonstrated that contrast enhancement correlated with areas of decreased cerebral blood volume [13]. However, the infarcted brain is not predictive of contrast extravasation or enhancement [22]. While elucidation of the etiology of CT hyperdensity is challenging, dual-energy CT (DECT) was clear on what constituted contrast. Phan et al. concluded on a series of 42 patients that DECT accurately differentiates between contrast and blood, but that metallic streak and calcifications could lead to identification failure [12]. This was supported by later studies [6,7,23].

Clinical Outcomes

Many of the studies reviewed focused on understanding and classifying the consequences of post-thrombectomy CT hyperdensities. Studies reviewed utilized the European Cooperative Acute Stroke Study II (ECASS) framework to classify images seen and communicate results. The ECASS trial classified cerebral hemorrhage after thrombolysis as hemorrhage infarct (HI) or parenchymal hemorrhage (PH) [24]. HI1 was defined as small petechiae along the margins of the infarct. HI2 were confluent petechiae in the infarcted area with no mass effect. PH1 was defined as a hematoma in less than 30% of the infarcted area with mild mass effect and PH2 as a hematoma in greater than 30% of the area with prominent mass effect. Although possible outcomes included resolution, infarct, and hemorrhagic transformation, Berger et al. concluded that only the PH2 cohort led to clinical deterioration [25]. Amans et al. concluded that contrast staining in the parenchyma was secondary to infarct and unlikely to convert to hemorrhage. He contended that the most relevant aspect of the hyperdensity was its effect clinically [5].

Song et al. reported that a contrast enhancement area ratio (CEAR) greater than 0.2 leads to malignant cerebral edema within 6 to 22 hrs. They also stressed that the calculation should not be the diagnostic criterion but rather that the extent of contrast staining be utilized as a predictive factor for malignant cerebral edema and poor prognosis [10].

Risk Factors in Patients That Lead to Post-procedure Hyperdensities

Due to the effects of type 2 parenchymal hemorrhagic (PH2) conversions, many studies identified pre-procedural risk factors for patients undergoing thrombectomy. These are summarized in Table 2.

**Table 2:** Pre-procedural risk factors for patients undergoing mechanical thrombectomy NIHSS: The National Institutes of Health Stroke Scale; TICI: thrombolysis in cerebral infarction.

Factors	Risk factors
Medical comorbidities [26,27]	Atrial fibrillation, diabetes mellitus, congestive heart failure, hypertension
Medications [28]	Antiplatelet therapy
Radiographic markers [27,29-33]	Infarct size greater than 1/3 of vascular territory, poor baseline CT (ASPECTS <=7) demonstrating brain edema, loss of grey white matter on CT, angiographic grade of poor collaterals, proximal MCA occlusion, reduced cerebral blood volume and flow on perfusion studies
Laboratory values [26-28,34,35]	Thrombocytopenia, hyperglycemia (>160), lower total cholesterol and low-density lipoprotein cholesterol, neutrophil to lymphocyte ratio > 3.89
Clinical factors [27,28,31]	Increased time to recanalization, transfer from outside facility, high NIHSS (>19)
Procedural factors [27,31,36]	Less than TICI 3 score, general anesthesia, higher thrombectomy maneuver counts

Thrombolytics activate plasminogen that lyses the fibrin clot, which increases the likelihood of hemorrhage. For patients already deemed higher risk, it has been suggested to avoid the use of intra-arterial thrombolytics [37,38]. Hassan et al. evaluated 329 patients and concluded that the number of stent retriever passes was not associated with the incidence of hemorrhagic transformation [26]. Kaesmacher et al. concluded, in his single-center study, that antiplatelet therapy was not related to increased risk of IPH and to accept that hemorrhagic infarcts and parenchymal hemorrhages do not always share the same risk factors or pathogenesis [27].

Neurologic Outcome

The studies listed in Table 3 address the clinical questions authors sought to evaluate including the impact of CT hyperdensities on neurological outcomes, malignant brain edema, symptomatic intraparenchymal hemorrhage, and areas of an infarct. 

**Table 3:** Authors and the clinical questions they studied BBB: blood-brain barrier.

Author	Clinical question
Sun [6]	What are the clinical and radiographic factors that contribute to optimal neurological outcomes?
Cabral [8]	How does hyperdensity (HD) correlate with final brain infarct?
Song [10]	Can contrast enhancement predict malignant cerebral edema?
Kim [29]	Can contrast extravasation predict symptomatic hemorrhage?
Amans [5]	Does contrast show the area of infarct?
Lummel [11]	What is the prognostic value of hyperattenuated intracerebral lesions?
Parrilla [37]	What is the clinical significance of HD after intra-arterial mechanical thrombectomy?
Nakano [15]	How often are early CT signs associated with BBB disruption and result in hemorrhagic transformations?
Jang [16]	How can we classify hyperdense lesions according to their morphologic features and what are the outcomes of those lesions?
Yoon [4]	What are the clinical consequences of contrast enhancement and contrast extravasation on CT scans?
Mericle [18]	Can a grading system be designed predicting outcomes after contrast extravasation?

Discussion

Terminology

There is no standardization in the utilization of the terms ‘contrast staining,’ ‘contrast enhancement,’ or ‘contrast extravasation.’ The terms are used interchangeably. While examining studies the use of terms ‘contrast enhancement’ and ‘contrast staining’ made the most sense in the setting of highlighting infarcted area and, as such, could be used interchangeably. The term ‘contrast extravasation’ was more likely used to describe the result of a persistent appearance on CT and thus included some form of hemorrhagic component. This could also be interpreted as describing a persistent mixed hyperdensity. The term ‘extravasation’ also seemed to be linked to describing areas of a more severe infarct. No matter the terminology, it appears the intent is to describe situations in which there may be a progression of a recognized CT hyperdensity to calling it a hemorrhage. It appears inherent that a temporal element or element of progression/severity/magnitude is associated with this terminology to better communicate this intention. In a new classification scheme, contrast staining/contrast enhancement would be limited to early post-MT scans where there is a very low likelihood of containing intermixed hemorrhage. ‘Contrast extravasation (CE)’ could then imply mixed hyperdensity in which there is a clear indication of the possibility of some degree of hemorrhage in addition to contrast (based again on accepted criteria and possibly even subjected to a staging protocol). The intent is to convey an increasing severity or magnitude of CE vs CS and progression from CS to CE, but not vice versa.

Lastly, use the ‘intraparenchymal hemorrhage (IPH)’, and then add a a suffix such as -C to indicate a IPH with the addition of contrast media within the body of the clot (IPH-C). This differentiation could be useful in follow-up exams, evaluation of contrast resolution, clot expansion/new hemorrhage, etc.

Imaging

The literature reviewed focused on CT imaging and determining ways to interpret post-procedural images. Other modalities were found to have fewer difficulties in making accurate interpretations. MRI DWI sequences are useful but are costly and time-consuming compared to CT. DECT can distinguish the difference between blood and contrast when standard definitions are applied. Unfortunately, DECT is not as readily available as CT or MR. A case could be made for that DECT be made a ‘requirement’ for thrombectomy-capable and comprehensive stroke centers. Conversely, would such an expense be justifiable if the overall difference in clinical outcomes is small? From a policy perspective, multiple factors would have to be considered before such a recommendation is made. Alternatively, hospitals are driven by competition, so marketing an advancement in imaging may allow DECT to become more prevalent. In the meantime, collaboration should occur on rectifying the challenges of interpretation with current CT/MR imaging.

Clinical Outcome

There is an association with the nature or degree of the contrast hyperdensity (simple staining vs extravasation) and potential clinical outcome variability (progression to worse clinical outcomes with possible formation of hemorrhagic transformation). This should provoke treating clinicians to follow their patients closely for possible conversion or clinical deterioration as poor outcomes are driven by type 2 intraparenchymal hemorrhages. 

For established large hemorrhages, the clinical outcome most likely is influenced not by the contrast but by the size, location, and evolution of the hemorrhage. If a matched study of post-infarction hemorrhage versus post-thrombectomy hemorrhage shows subtle differences in outcomes, this would call into question pathologies associated with both the procedure and the contrast used during the procedure. No study looking at this has been proposed yet.

Studies on thrombectomy maneuvers are conflicting but suggest clinical outcomes may be related to the number of passes as well as the final thrombolysis in cerebral infarction (TICI) score [26,36]. TICI 3 grade is associated with better outcomes and a lesser chance of contrast extravasation and IPH type 2 when compared to lesser TICI scores. Given the potential noxious effect of contrast, studies quantifying the amount of contrast staining/contrast extravasation/contrast in a clot with effect on outcome may yield interesting results. No such study has yet been designed.

Summary and Future Directions

Our review regarding post-thrombectomy contrast staining/extravasation led to two major conclusions and a substantive inference. First is the need to standardize terminology, greatly increasing our ability to compare and assess data across studies. Second is the need for better qualification/quantification regarding imaging studies, risk factors, and clinical correlates. Current literature supports a role for mixed density contrast/blood extravasation as being a possible hinge point on treatment decisions (continue paradigms as if there is no IPH vs. alter paradigms for a patient with IPH). What is not clear is at what point does a small amount of blood/contrast represents a true threat of transformation to full IPH. With the standardization of post-MT CT findings, appropriate data collection, follow-up, or possibly with the increased use of DECT, several avenues of study could be generated that may indeed lead to the discovery of pre-IPH risk factors that may alter standards of care and improve clinical outcomes. Currently, there is no proven intervention to prevent hemorrhagic transformation. However, reduction of MMP-2 and administration of deferoxamine, estrogen, or cilostazol are being studied [39]. Early identification and recognition of contrast staining/extravasation may be a key player in the management of these novel agents.

## Conclusions

Based on our review, several conclusions could be drawn as summarized below. The authors suggest pursuing a standardization process. A universally accepted standard radiographic and nomenclature categorization scheme for post-mechanical thrombectomy CT hyperdensities should be applied, then rapidly associated with pre-identified periprocedural risk factors/outcomes. A standardized follow-up protocol involving imaging and clinical evaluation is recommended as it will allow clinicians to discover the relationships these hyperdensities have with various clinical stroke states. If adjustments to patient care are then made, this may impact clinical outcomes.

While many variables exist that may influence individual outcomes, it is the authors’ opinion that inclusion of contrast staining after MT as a data point be considered relevant since there is no harm in using the data to determine if a predictive value and/or outcome influence exists (one does not intentionally cause contrast staining after MT solely to study its effect; it occurs as a result of procedure or progression of infarction).

Patients who would benefit from post-procedural anticoagulation or antiplatelet therapy to prevent reocclusion/progression are sometimes delayed until a final diagnosis of IPH can be made. Therefore, proper diagnosis of a hyperdensity is critical. Stroke centers should continue to invest in technologies that improve patient safety, reduce errors or missed diagnoses, and strive toward decreasing morbidity and mortality. Based on our review, useful information may exist that may impact clinical outcomes. We postulate the next best move forward in the study of this phenomenon is to standardize both terminology and process. Once achieved, the potential for relevant avenues of clinical study would emerge.
